# Nomogram for soiling prediction in postsurgery hirschsprung children: a retrospective study

**DOI:** 10.1097/JS9.0000000000000993

**Published:** 2023-12-19

**Authors:** Pei Wang, Erhu Fang, Xiang Zhao, Jiexiong Feng

**Affiliations:** Department of Pediatric Surgery, Tongji Hospital of Tongji Medical College of Huazhong University of Science and Technology; Hubei Clinical Center of Hirschsprung Disease and Allied Disorders, Wuhan, People’s Republic of China

**Keywords:** HSCR, nomogram, prediction model, soiling

## Abstract

**Purpose:**

The aim of this study was to develop a nomogram for predicting the probability of postoperative soiling in patients aged greater than 1 year operated for Hirschsprung disease (HSCR).

**Materials and methods:**

The authors retrospectively analyzed HSCR patients with surgical therapy over 1 year of age from January 2000 and December 2019 at our department. Eligible patients were randomly categorized into the training and validation set at a ratio of 7:3. By integrating the least absolute shrinkage and selection operator [LASSO] and multivariable logistic regression analysis, crucial variables were determined for establishment of the nomogram. And, the performance of nomogram was evaluated by C-index, area under the receiver operating characteristic curve, calibration curves, and decision curve analysis. Meanwhile, a validation set was used to further assess the model.

**Results:**

This study enrolled 601 cases, and 97 patients suffered from soiling. Three risk factors, including surgical history, length of removed bowel, and surgical procedures were identified as predictive factors for soiling occurrence. The C-index was 0.871 (95% CI: 0.821–0.921) in the training set and 0.878 (95% CI: 0.811–0.945) in the validation set, respectively. And, the AUC was found to be 0.896 (95% CI: 0.855−0.929) in the training set and 0.866 (95% CI: 0.767−0.920) in the validation set. Additionally, the calibration curves displayed a favorable agreement between the nomogram model and actual observations. The decision curve analysis revealed that employing the nomogram to predict the risk of soiling occurrence would be advantageous if the threshold was between 1 and 73% in the training set and 3–69% in the validation set.

**Conclusion:**

This study represents the first efforts to develop and validate a model capable of predicting the postoperative risk of soiling in patients aged greater than 1 year operated for HSCR. This model may assist clinicians in determining the individual risk of soiling subsequent to HSCR surgery, aiding in personalized patient care and management.

## Introduction

Highlights

- This is the first study to establish and validate a model for predicting the risk of soiling in patients aged greater than 1 years operated for Hirschsprung disease.
- In this work, we determined that surgical history, length of removed bowel, and surgical procedures were the main factors associated with soling in patients with Hirschsprung disease.
- The developed predictive model will be valuable in identification and stratification of patients following operative management.

Hirschsprung disease (HSCR), which affects ~1 per 5000 children, is a common congenital deformity that caused by the incomplete craniocaudal migration of enteric neural crest cells during embryological development^1,2^. Presently, the optimal treatment for HSCR is surgery to remove the aganglionic bowel^3^. With the significant progress have been achieved in the management of HSCR, the quality of life of patients has been greatly improved. However, certain individuals still experienced various postoperative complications, especially the soiling, remains affecting the quality of life of HSCR patients^4,5^.

Soiling, characterized by the involuntary leakage of stool that requires a change of underwear or diapers, is a prevalent complication following the surgery of HSCR^6,7^. The currently available reports on the prevalence of soiling following the surgery for HSCR vary widely^6^. A new systematic review indicated that the prevalence of soiling range from 4.1 to 54.5% after transanal endorectal pull-through (TEPT) and from 0 to 37.5% after laparoscopic-assisted TEPT (LAPT)^8^. In theory, the incidence of postoperative soiling should be low for HSCR patients undergoing surgical procedures, as patients are born with a fully developed anal anatomy, including intact sensation and functional voluntary sphincters. Additionally, a well-experienced surgeon and the availability of surgical techniques were responsible for the function of the anal canal, thus to maintain regular intestinal function postoperatively^9^. At present, the precise mechanism underlying soiling in patients with HSCR remains incompletely understood, although it may be related to several factors, such as abnormal anal canal sensation, inadequate sphincter control, and the degree of bowel dysmotility^10^.

In the context of soiling in patients with HSCR, previous studies mainly focus on individuals who underwent surgery before the age of 1 year^6,11^. Nevertheless, it is a well-established fact that infants under the age of one year possess inadequate bowel control capabilities^12^, making it difficult to assess the condition of soiling. Simultaneously, soiling can result from various factors, including external causes like medications as well as unknown elements related to certain conditions such as Down syndrome^13,14^. Importantly, infants are especially susceptible to these various soiling factors. Therefore, we first determined factors associated with soiling occurrence in patients over the age of one who underwent surgery for HSCR.

This study is the first to establish and validate a model for predicting the risk of soiling in patients with HSCR following surgical therapy beyond the age of 1 year. We proposed that this nomogram be readily accessible to clinicians, enabling them to easily estimate the risk of soiling in their patients.

## Methods

### Patients

This retrospective study was conducted on patients diagnosed with HSCR who underwent surgical treatment at our hospital department from January 2000 to December 2019. This research study was reviewed and approved by the Ethics Committee at Hospital, waiving the requirement for informed consent in alignment with the Declaration of Helsinki. The diagnosis of HSCR was established through preoperative examinations, which included barium enema, anorectal manometry, and suction rectal biopsy. Additionally, postoperative pathological examination was conducted to further confirm the diagnosis. All operations were performed in accordance with the standard procedures by experienced surgical groups in our department.

### Study protocol

The work was approved by the Ethics Committee and registered on Clinicaltrial.gov. It was conducted and reported in line with the strengthening the reporting of cohort, cross-sectional and case–control studies in surgery (STROCSS) criteria^15^.

### Definition of soiling

**Figure 1:**
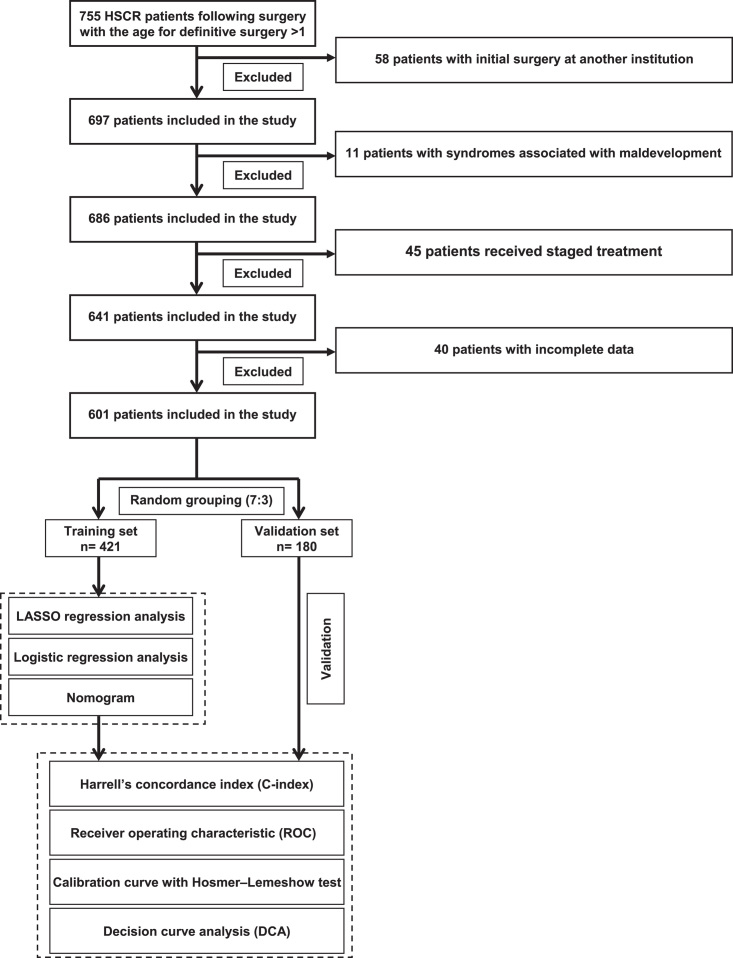
Flowchart of this study.

This study refers to the diagnostic criteria for soiling as described in the published literature^16,17^. Based on the previously established criteria and clinical experience, patients who experienced soiling persisting for more than 6 months following surgery were ultimately identified for inclusion in this study. In this study, patients with Down’s syndrome, and other established syndromes potentially contributed to maldevelopment were excluded as previous described^18^. In addition, patients who had their initial surgery at another institution were excluded from the study, as well as any participants received staged treatment or had missing data related to soiling. A study flow chart is illustrated in Figure 1.

### Data collection and potential predictors

In this study, we collected the clinical information about patients from a thorough review of relevant literatures and clinical judgement. These data included the age for definitive surgery, sex, weight at operation, premature delivery history, family history, meconium, duration of constipation, conservative treatment, surgical history, surgical techniques, length of removed bowel, surgical procedure, and soiling status. The surgical techniques included LAPT, laparotomy-assisted endorectal pull-through (LEPT), and TEPT. Length of removed bowel included left colectomy, subtotal ectomy, and total colectomy. Surgical procedures were categorized as Soave, Heart-Shaped, Rebbein, Duhamel, and others. Data with a deletion rate of greater than or equal to 20% were excluded from the analysis. The trimming method was introduced to address with abnormal values and a multiple imputation method was employed to interpolate missing data^19,20^.

### Logistic regression analysis and nomogram model development

A total of 601 patients were randomly assigned into training and validation set in a 7:3 ratio as previous reported^21,22^. No significant differences were observed in demographic and clinical characteristics between the training and validation sets. To determine potential predictive factors, the training set was subjected to LASSO regression analysis, which effectively eliminated several irrelevant or multicollinearity independent variables to reduce high-dimensional data^23^. Then, the multivariable logistic regression analysis was applied to determine the variables of soiling in patients with HSCR following surgery over one year of age. Finally, a nomogram was created based on the training set and validated in the validation set.

### Apparent performance of the nomogram

Discrimination and calibration were employed to assess and test the predictive accuracy of the established model^24^. Harrell’s concordance index (C-index) and the receiver operating characteristic (ROC) curve (AUC) were generated to estimate the discrimination of the nomogram^25^. A C-index close to 1 indicates a high level of predictive ability of the model. Additionally, an AUC greater than 0.80 indicates relatively good discrimination^26,27^. The calibration of the nomogram was accompanied with the Hosmer–Lemeshow test were developed to determine the consistency between the predicted and observed occurrence of soiling^28^. Lastly, DCA was performed to evaluate clinical practicability of the nomograms^29^. Both discrimination and calibration were assessed by bootstrapping with 1000 resamples.

### Statistical analysis

For parameters with continuous variables, the normal distribution was expressed as mean±SD, and the skewed distribution was presented as median [M] and quartile range [Q25−Q75]. Continuous data between two groups were compared using the independent *t*-test. The unequal variance *t*-test was used to compare data between groups with unequal variances. In cases of skewed distribution, the Mann–Whitney *U* test was employed. Furthermore, categorical data were compared using χ^2^ or Fisher’s exact test. Statistical analysis was performed using R Software v.4.0.2 (The R Project for Statistical Computing, www.r-project.org) with the ‘rms’ package utilized for logistic regression analysis and nomogram construction. A two-sided *P*-value less than 0.05 was considered statistically significant.

## Results

### Patients’ characteristics

As depicted in Table 1, a total of 601 eligible patients were included in this study. The entire cohort was randomly assigned to a training set (*n*=421) and a validation set (*n*=180). Among them, 97 patients experienced postoperative soiling, with 68 cases in the training set and 29 cases in the validation set. Statistical analysis revealed no significant differences between the training set and validation set (*P*>0.05). The baseline characteristics of patients were given in Tables 1–2.

**Table 1:** Baseline characteristics between the soiling and nonsoiling groups.

	Soiling		
Characteristic	No (*n*=504) M (Q25, Q75)/*N* (%)	Yes (*n*=97) M (Q25, Q75)/*N* (%)	*P*
Age for definitive surgery (year)	3.00 (1.33, 5.00)	2.67 (1.46, 4.71)	0.692
Sex			
Male	296 (58.7)	50 (51.5)	0.190
Female	208 (41.3)	47 (48.4)	
Weight at operation (kg)	13.00 (10.00, 17.00)	13.00 (10.00, 15.75)	0.193
Premature delivery history			
No	493 (97.8)	95 (97.9)	1.00
Yes	11 (2.2)	2 (2.1)	
Family history			
No	502 (99.6)	97 (100)	1.00
Yes	2 (0.40)	0 (0)	
Meconium (h)			
≤24	256 (50.8)	45 (46.4)	0.427
＞24	248 (49.2)	52 (53.6)	
Duration of constipation (year)	1.67 (0.92, 3.29)	1.75 (0.92, 3.00)	0.958
Conservative treatment			
No	192 (38.1)	46 (47.4)	0.085
Yes	312 (61.9)	51 (52.6)	
Surgical history (times)			
1	462 (91.7)	33 (34.0)	<0.0001
>1	42 (8.3)	64 (66.0)	
Surgical techniques			
LAPT	220 (43.6)	31 (32.0)	0.065
LEPT	257 (51.0)	62 (63.9)	
TPET	27 (5.4)	4 (4.1)	
Length of removed bowel			
left colectomy	299 (59.3)	25 (25.8)	<0.0001
subtotal colectomy	184 (36.5)	57 (58.7)	
total colectomy	21 (4.2)	15 (15.5)	
Surgical procedures			
Soave	199 (39.5)	199 (39.5)	<0.0001
Heart-shaped	243 (48.2)	20 (20.6)	
Rebbein	44 (8.7)	13 (13.4)	
Duhamel	10 (2.0)	4 (4.1)	
Others	8 (1.6)	2 (2.1)	

LAPT, laparoscopic-assisted pull-through; LEPT, laparotomy-assisted endorectal pull-through; TEPT, transanal endorectal pull-through.

**Table 2:** Baseline characteristics of participants in training and validation set.

Characteristic	Total (*n*= 601)	Training set (*n*=421) M (Q25, Q75)/*N* (%)	Validation set (*n*=180) M (Q25, Q75)/*N* (%)	*P*
Age for definitive surgery (year)	2.92 (1.33, 5.00)	2.92 (1.33, 5.00)	2.96 (1.52, 5.00)	0.568
Sex				
Male	346 (57.6)	245 (58.2)	101 (56.1)	0.636
Female	255 (42.4)	176 (41.8)	79 (43.9)	
Weight at operation ( kg)	13.00 (10.00, 17.00)	13.00 (10.00, 17.00)	13.00 (10.00, 16.88)	0.875
Premature delivery history				
No	587 (97.7)	412 (97.9)	175 (97.2)	0.634
Yes	14 (2.3)	9 (2.1)	5 (2.8)	
Family history				
No	599 (99.7)	419 (99.5)	180 (100)	1.000
Yes	2 (0.3)	2 (0.5)	0 (0)	
Meconium (h)				
≤24	301 (50.1)	207 (49.2)	94 (52.2)	0.493
＞24	300 (49.9)	214 (50.8)	86 (47.8)	
Duration of constipation (year)	1.67 (0.92, 3.17)	1.58 (0.92, 3.25)	1.83 (0.92, 3.13)	0.772
Conservative treatment				
No	238 (39.6)	168 (39.9)	70 (38.9)	0.816
Yes	363 (60.4)	253 (60.1)	110 (61.1)	
Surgical history (times)				
1	499 (83.0)	342 (81.2)	157 (87.2)	0.073
>1	102 (17.0)	79 (18.8)	23 (12.8)	
Surgical techniques				
LAPT	251 (41.8)	180 (42.8)	71 (39.5)	0.244
LEPT	319 (53.1)	216 (51.3)	103 (57.2)	
TPET	31 (5.1)	25 (5.9)	6 (3.3)	
Length of removed bowel				
Left colectomy	324 (53.9)	223 (53.0)	101 (56.1)	0.195
Subtotal colectomy	241 (40.1)	168 (39.9)	73 (40.6)	
Total colectomy	36 (6.0)	30 (7.1)	6 (3.3)	
Surgical procedures				
Soave	257 (42.8)	185 (43.9)	72 (40.0)	0.909
Heart-shaped	263 (43.8)	180 (42.8)	83 (46.1)	
Rebbein	57 (9.5)	40 (9.5)	17 (9.4)	
Duhamel	14 (2.3)	9 (2.1)	5 (2.8)	
Others	10 (1.6)	7 (1.7)	3 (1.7)	
Soiling				
No	504 (83.9)	353 (83.8)	151 (83.9)	0.990
Yes	97 (16.1)	68 (16.2)	29 (16.1)	

LAPT, laparoscopic-assisted pull-through; LEPT, laparotomy-assisted endorectal pull-through; TEPT, transanal endorectal pull-through.

### Identification of predictive factors

**Figure 2:**
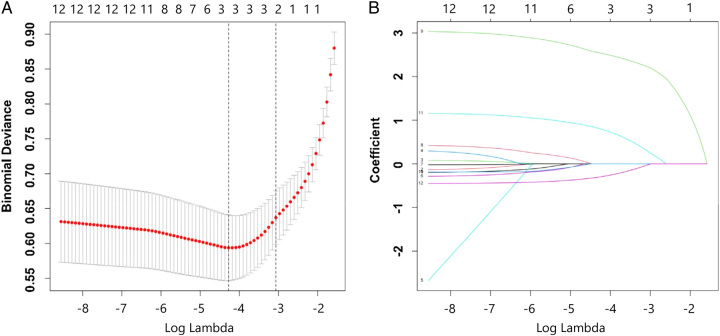
[A] The optimal parameter [λ] selection in the LASSO model employed fivefold cross-validation using a minimum criteria approach. The optimal values of λ are represented by dotted vertical lines. Among these values, λ=0.012, corresponding to a logarithm of λ equal to −4.422, was selected as the optimal choice. [B] LASSO coefficient profiles of 12 clinical features. The plot was created using a logarithmic scale for the lambda values. A vertical line was added to indicate the lambda value selected through fivefold cross-validation. This optimal lambda value led to the identification of three features with nonzero coefficients.

Given the multitude of variables involved, a two-step approach was employed to filter the clinical features. Firstly, a preliminary screening was performed using the LASSO regression to identify potential predictors, ensuring avoidance of overfitting and enhancement of the model’s robustness (Figure 2A and B). As a result, three candidate predictors were determined, including operation history, length of removed bowel, and surgical procedures. Following this, these three features predictors were subjected to multivariate logistic regression. The results demonstrated that surgical history >1 (OR=22.89, 95% CI: 11.21–49.08, *P*<0.001), subtotal colectomy (OR=3.39, 95% CI: 1.60–7.45, *P*=0.002), total colectomy (OR=11.60, 95% CI: 3.30–42.78, *P*<0.001), Rehbein anastomosis (OR=0.27, 95% CI: 0.09–0.77, *P*=0.017), Heart−Shaped anastomosis (OR=0.33, 95% CI: 0.14–0.74, *P*=0.008), other anastomosis (OR=0.03, 95% CI: 0.001–0.37, *P*=0.011) were independent predictors for soiling in HSCR following surgery over 1 year of age. The multivariate analyses of soiling in the training set are listed in Table 3.

**Table 3:**
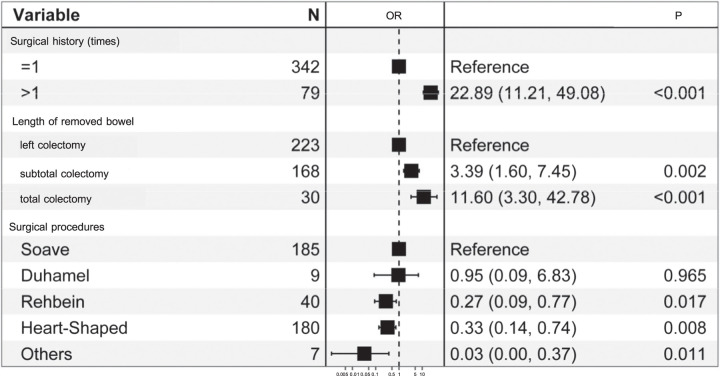
The prediction model with multivariate logistic regression.

### Development of an individualized prediction model

**Figure 3:**
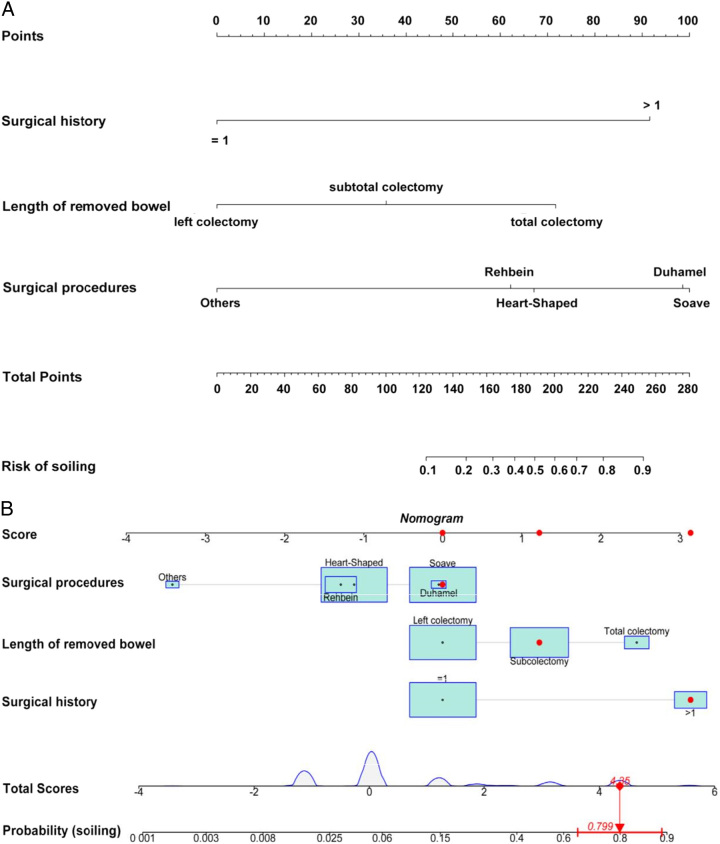
[A] Nomogram with surgical history, length of removed bowel, and surgical procedures predicts the probability of soiling. [B] The dynamic nomogram reveals the surgical outcomes in a patient with HSCR. This patient experienced multiple surgeries [surgical history >1], with the most recent procedure involving subtotal colectomy and Soave anastomosis. The estimated probability of soiling for this case is 0.799.

A nomogram was established by incorporating the aforementioned crucial clinical features. The nomogram revealed the relative contributions of each factor to the risk of soiling. According to the nomogram, Soave anastomosis was identified as the strongest predictor of soiling, followed by Duhamel anastomosis, surgical history (>1), total colectomy, Heart−Shaped anastomosis, Rehbein anastomosis, and subcolectomy. Meanwhile, the nomogram also highlighted protective factors, including surgical history (=1), left colectomy, and other surgical procedures (Fig. 3).

### Predictive model validation

#### Discrimination

**Figure 4:**
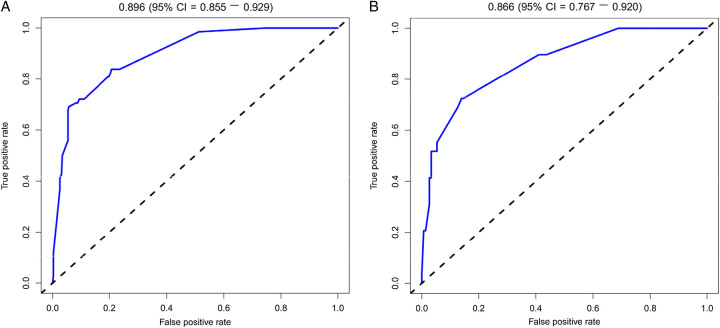
The area under the receiver operating characteristic curve [AUC] for the discrimination of the model. [A] The training set, 0.896 [95% CI: 0.855−0.929]. [B] The validation set, 0.866 [95% CI: 0.767−0.920].

For the sample, the C-index was 0.877 (95% CI: 0.828–0.926) in the training set and 0.878 (95% CI: 0.811–0.945) in the validation set, indicating that the model presented a good discriminative power. Additionally, the AUC values were determined to evaluate the discrimination of the nomogram. In the training set, the AUC for the nomogram to predict soiling was 0.896 (95% CI: 0.855−0.929). In the validation set, the AUC remained as high as 0.866 (95% CI: 0.767−0.920), further supporting the robust discriminative ability of the model (Fig. 4).

#### Calibration of the predictive model

**Figure 5:**
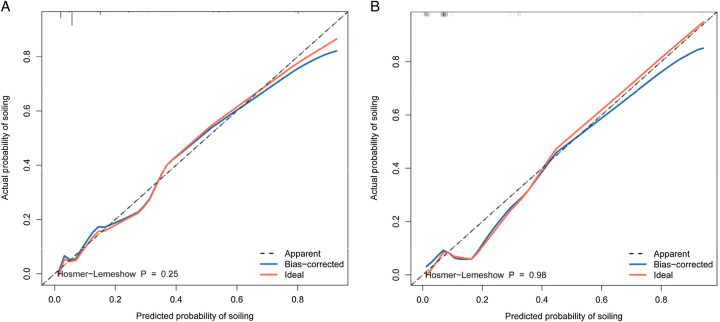
Calibration curves for the predicting probability of soiling in the training set [A] and [B] in the validation set, all *P*-value >0.05 in the Hosmer–Lemeshow test suggested an agreement between the predicted probabilities and observed outcomes.

Furthermore, the calibration curve presented no difference between the predicted and actual probabilities of soiling occurrence in the training set (χ^2^=10.11, df=8, *P*=0.257) and the validation sets (χ^2^=2.046, df=8, *P*=0.978) (Fig. 5).

#### Clinical use

**Figure 6:**
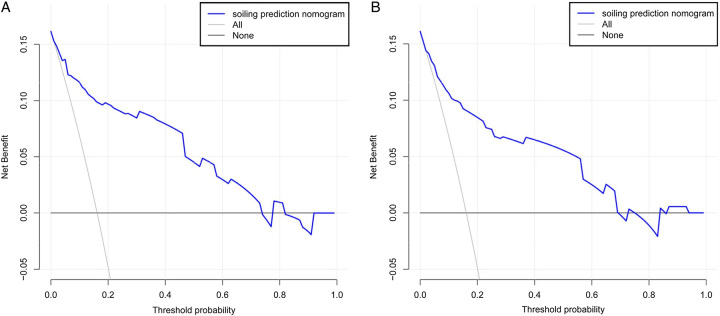
Decision curve analysis [DCA] for the soiling nomogram. The black line represents the assumption of no patient having soiling, while the gray line assumes that all patients experienced soiling. The blue line corresponds to the risk nomogram. The analysis was conducted on both the training set [A] and the validation set [B].

Moreover, the result of DCA indicated that the nomogram could yield significant net benefits for the patients experiencing soiling. These benefits were observed within a risk threshold probability range of 1 to 73% in the training set and 3–69% in the validation set (Fig. 6).

## Discussion

Despite significant advancements have been achieved in the management of HSCR in recent years, the occurrence of postoperative complications, particularly soiling, remains prevalent. Following surgery treatment, patients with HSCR often experience different degrees of soiling^6,17^. The occurrence of soiling can lead to various physical and psychological problems, ultimately exerting a negative impact on an individual’s quality of life^30^. It has been suggested the etiology of postsurgical soiling in children can be attributed to three main factors: impaired sphincter function, dysfunction of the rectal reservoir, and diminished anal canal sensation^10,31^. And, impairment of any of these factors can compromise bowel control and result in the development of soiling^6^. Furthermore, additional factors such as the patient’s age at the time of surgery and the type of surgical procedure performed also play a role in determining bowel function^6,17^.

Several studies have shown a correlation between postoperative soiling and the age of the patient undergoing surgical management. Previously, a study showed that some infants experienced a dramatic reduction in stool frequency after surgery for HSCR^32^. However, the specific mechanism behind this age-related improvement remains unclear. Moreover, another study aimed to investigate the outcomes of patients with HCSR who underwent surgery prior to their first birthday was conducted, which demonstrated the differences of bowel functional decreased with age^6^. Nevertheless, the correlation between soiling and patients aged greater than 1 years operated for HSCR has never been thoroughly evaluated. With this in mind, we developed a nomogram for the first time to assess the risk of soiling in patients aged greater than 1 years operated for HSCR.

The Nomogram is a widely used prediction model in both oncologic and nononcologic diseases^33,34^. And, nomogram is established by integrating key factors to visualize the probability of clinical outcomes. A well-developed nomogram is a popular decision making tool that can be readily available to clinicians. Currently, there is an absence of published nomograms that predict the occurrence of soiling in HSCR patients postoperatively. This is the first study to establish and validate a model for predicting the risk of soiling in patients aged greater than 1 years operated for HSCR. In this work, we determined that surgical history, length of removed bowel, and surgical procedures were the main factors associated with soling in patients with HSCR. A predictive model of soiling was developed based on the three factors, demonstrating favorable discrimination, calibration, and clinical utility. These findings indicating that the model is valuable in identification and stratification of patients following operative management, which helps clinicians make an early intervention to reduce or avoid the occurrence of soiling.

Several studies have provided evidence to support the aforementioned three soiling-related risk factors. Prior studies have demonstrated that preserving the internal sphincter and preventing spur formation during surgery can decrease the risk of postoperative soiling^35,36^. And, proper sensory function relies on an undamaged anal canal, enabling distinction between solid, liquid, and gaseous rectal contents^37,38^. In the surgery, the anal canal may suffer damage if the distal end of the intraluminal dissection performed from above breaches into the anal canal^9^. Consequently, successive surgery and excessive surgical resections may impair the function of sphincter and the intact of anal canal, contributing to postoperative soiling.

Furthermore, the choice of surgical procedure also influences the development of postoperative soiling. For example, available researches indicated HSCR patients treated with the Duhamel procedure present with reduced postoperative soiling, as this surgical approach appears to minimize trauma to the anal canal^39^. The rectal sensation relies on perceiving stretching of the rectal walls and input from the specialized transitional epithelium demarcated by the dentate line. For HSCR pull-through procedures, the anastomosis should be performed 5–15 mm proximal to the dentate line^40^. Disrupting the dentate line during surgery may impair rectal awareness, leading to potential postoperative soiling. Heart-shaped anastomosis as a surgical procedure has been widely introduced to treat HSCR patients in our center^41^. The heart-shaped anastomosis surgical technique has several advantages compared to other methods like the Duhamel procedure. By removing only the posterior internal sphincter while preserving the anterior sphincter, it helps avoid complications like internal sphincter spasms and soiling issues after surgery. Additionally, since the rectocolic anastomosis is performed end-to-end, there is no risk of a blind rectal pouch or septum forming as can happen with the Duhamel procedure. The heart shape of the anastomosis also makes strictures and constipation less likely compared to other procedures^18,42^. Overall, the specific design of the heart-shaped anastomosis appears to minimize risks of common postsurgical problems like soing, strictures, and constipation.

In clinical practice, the developed model could estimate the probability of postoperative soiling in patients aged greater than 1 year operated for HSCR. Consequently, this model may assist clinicians in identifying patients who are at risk of suffering soiling subsequent to HSCR surgery, aiding in personalized patient care and management. As shown in Figure 3B, the patient experienced multiple surgeries (surgical history >1), with the most recent procedure involving subtotal colectomy and Soave anastomosis. And, the estimated probability of soiling is 0.799. These results indicate that previous surgical history and length of removed bowel significantly impacts the subsequent occurrence of soiling, which is in lined with previous studies^43,44^. Accordingly, a comprehensive assessment involving a detailed history and clinical examination is imperative preceding additional operative interventions. Furthermore, in the case of high-risk patients, clinicians may choose more conservative surgical approaches and increased postoperative monitoring. In addition, utilizing the model results, clinicians can engage in comprehensive discussions of surgical risks with patients and families and manage patient expectations regarding surgical outcomes. These attributes may facilitate more satisfactory treatment results and cost-effective medical practices.

## Limitations

There are some limitations in this work. First, due to the nature of retrospective study^45,46^, this study is inherently limited in its ability to establish causation. Moreover, some potentially factors influenced soiling, such as detailed dietary habits, postoperative care/compliance, and patient genetics are not available in our retrospective data, as we were constrained to the data available in the medical records. We believe that further prospective studies are needed to establish causation, validate the associations observed in our retrospective analysis. In addition, our model development and validation was done internally as the availability of data, which allowed us to fully optimize the model in a relatively controlled environment^47^ but limited the generalizability of the model to other institutions. As is well-established, external validation offers a more rigorous assessment of model robustness, while noise or biases in external validation datasets may obscure the true model performance discovered through internal validation^48,49^. Finally, this study was conducted with patients from a single medical institution, it remains challenging to fully eliminate the selection bias and information bias associated with single-site samples. Hence, conducting multicenter and prospective cohort studies is imperative for a more thorough exploration^50,51^.

There are some limitations in this work. First, several potential factors, such as dietary habits, were not available in our data. In addition, it is important to acknowledge that the study might be limited to specific geographical areas. Therefore, the generalizability of the results to other regions necessitates further validation through the inclusion of data from external cohorts.

## Conclusion

To the best of our knowledge, this is the first study to develop and verify a nomogram for predicting the risk of soiling in patients aged greater than 1 year operated for HSCR. Our model, which integrates surgical history, length of removed bowel, and surgical procedures was verified internally as a useful tool for risk assessment. The developed predictive model will be valuable in identification and stratification of patients following operative management.

## Ethical approval

This study was approved by the Ethical Committee of Tongji Medical College of Huazhong University of Science and Technology (Ethical Committee S108) and conducted under the guidance of the Declaration of Helsinki.

## Consent

Written informed consent was not relevant to this manuscript.

## Sources of funding

This work is funded by the National Natural Science Foundation of China (81873541, Jiexiong Feng) and Tongji Hospital (2019YBKY026, Jiexiong Feng), and National Natural Science Foundation of China (82003124, Erhu Fang).

## Author contribution

P.W.: data curation, resources, investigation, and writing – original draft; E.F.: conceptualization, methodology, software, formal analysis, and writing – review and editing; X.Z.: visualization, investigation, and validation; J.F.: conceptualization, funding acquisition, resources, supervision, and writing – review and editing. All the authors contributed to the article and approved the submitted version.

## Conflicts of interest disclosure

The authors declare no conflicts of interest.

## Research registration unique identifying number (UIN)

1. Name of the registry: Clinicaltrials.gov.
2. Unique identifying number or registration ID: NCT06025188.
3. Hyperlink to specific registration: https://clinicaltrials.gov/ct2/show/NCT06025188.

## Guarantor

Jiexiong Feng.

## Data availability statement

The authors conform that the data supporting the findings of this study are available within the article and its supplementary materials. Also, any extra data of this study are available on request from the corresponding author.

## Provenance and peer review

The paper was not invited.

## References

[1] Fadista J Lund M Skotte L . Genome-wide association study of Hirschsprung disease detects a novel low-frequency variant at the RET locus. Eur J Hum Genet 2018;26:561–569. 29379196 10.1038/s41431-017-0053-7 PMC5891499

[2] Karim A Tang CS Tam PK . The emerging genetic landscape of Hirschsprung disease and its potential clinical applications. Front Pediatr 2021;9:638093. 34422713 10.3389/fped.2021.638093 PMC8374333

[3] Pan W Rahman AA Stavely R . Schwann cells in the aganglionic colon of Hirschsprung disease can generate neurons for regenerative therapy. Stem Cells Transl Med 2022;11:1232–1244. 36322091 10.1093/stcltm/szac076 PMC9801298

[4] Huang WK Li XL Zhang J . Prevalence, risk factors, and prognosis of postoperative complications after surgery for Hirschsprung disease. J Gastrointest Surg 2018;22:335–343. 28956279 10.1007/s11605-017-3596-6

[5] Soret R Schneider S Bernas G . Glial cell-derived neurotrophic factor induces enteric neurogenesis and improves colon structure and function in mouse models of Hirschsprung disease. Gastroenterology 2020;159:1824–1838. 32687927 10.1053/j.gastro.2020.07.018

[6] Oh C Youn JK Han JW . The patients with Hirschsprung’s disease who underwent pull-through at age less than 1 year: longitudinal bowel function. World J Surg 2020;44:2426–2439. 32161985 10.1007/s00268-020-05474-6

[7] Chantakhow S Khorana J Tepmalai K . Alterations of gut bacteria in Hirschsprung disease and Hirschsprung-associated enterocolitis. Microorganisms 2021;9:2241. 34835367 10.3390/microorganisms9112241 PMC8623574

[8] Celtik U Yavuz I Ergün O . Transanal endorectal or transabdominal pull-through for Hirschsprung’s disease; which is better? A systematic review and meta-analysis. Pediatr Surg Int 2023;39:89. 36692536 10.1007/s00383-023-05378-1

[9] Levitt MA Martin CA Olesevich M . Hirschsprung disease and fecal incontinence: diagnostic and management strategies. J Pediatr Surg 2009;44:271–277. 19159755 10.1016/j.jpedsurg.2008.10.053

[10] Saadai P Trappey AF Goldstein AM . Guidelines for the management of postoperative soiling in children with Hirschsprung disease. Pediatr Surg Int 2019;35:829–834. 31201486 10.1007/s00383-019-04497-y

[11] Taguchi T Obata S Ieiri S . Current status of Hirschsprung’s disease: based on a nationwide survey of Japan. Pediatr Surg Int 2017;33:497–504. 28058486 10.1007/s00383-016-4054-3

[12] Benninga MA Faure C Hyman PE . Childhood functional gastrointestinal disorders: neonate/toddler. Gastroenterology 2006;130:1519–1526. 16678565 10.1053/j.gastro.2005.11.065

[13] Bharucha AE Pemberton JH Locke GR III . American Gastroenterological Association technical review on constipation. Gastroenterology 2013;144:218–238. 23261065 10.1053/j.gastro.2012.10.028 PMC3531555

[14] Schill EM Wright CM Jamil A . Down syndrome mouse models have an abnormal enteric nervous system. JCI Insight 2019;5:e124510. 30998504 10.1172/jci.insight.124510 PMC6629165

[15] Mathew G Agha R for the STROCSS Group . STROCSS 2021: strengthening the reporting of cohort, cross-sectional and case-control studies in surgery. Int J Surg 2021;96:106165. 34774726 10.1016/j.ijsu.2021.106165

[16] Neuvonen MI Kyrklund K Rintala RJ . Bowel function and quality of life after transanal endorectal pull-through for hirschsprung disease: controlled outcomes up to adulthood. Ann Surg 2017;265:622–629. 28169931 10.1097/SLA.0000000000001695

[17] Gunadi Monica Carissa Stevie T . Long-term functional outcomes of patients with Hirschsprung disease following pull-through. BMC Pediatr 2022;22:246. 35505310 10.1186/s12887-022-03301-6 PMC9063042

[18] Xiong X Chen X Wang G . Long term quality of life in patients with Hirschsprung’s disease who underwent heart-shaped anastomosis during childhood: a twenty-year follow-up in China. J Pediatr Surg 2015;50:2044–2047. 26423683 10.1016/j.jpedsurg.2015.08.027

[19] Tam S Tsao MS McPherson JD . Optimization of miRNA-seq data preprocessing. Brief Bioinform 2015;16:950–963. 25888698 10.1093/bib/bbv019 PMC4652620

[20] Austin PC White IR Lee DS . Missing data in clinical research: a tutorial on multiple imputation. Can J Cardiol 2021;37:1322–1331. 33276049 10.1016/j.cjca.2020.11.010 PMC8499698

[21] Groos D Adde L Aubert S . Development and validation of a deep learning method to predict cerebral palsy from spontaneous movements in infants at high risk. JAMA Netw Open 2022;5:e2221325. 35816301 10.1001/jamanetworkopen.2022.21325 PMC9274325

[22] Chen Q Pan T Wang YN . A coronary CT angiography radiomics model to identify vulnerable plaque and predict cardiovascular events. Radiology 2023;307:e221693. 36786701 10.1148/radiol.221693

[23] Hu JY Wang Y Tong XM . When to consider logistic LASSO regression in multivariate analysis? Eur J Surg Oncol 2021;47:2206. 33895026 10.1016/j.ejso.2021.04.011

[24] Mejia OAV Borgomoni GB Palma Dallan LR . Quality improvement program in Latin America decreases mortality after cardiac surgery: a before-after intervention study. Int J Surg 2022;106:106931. 36126857 10.1016/j.ijsu.2022.106931

[25] Yu A Li Y Zhang H . Development and validation of a preoperative nomogram for predicting the surgical difficulty of laparoscopic colectomy for right colon cancer: a retrospective analysis. Int J Surg 2023;109:870–878. 36999773 10.1097/JS9.0000000000000352 PMC10389525

[26] Mandrekar JN . Receiver operating characteristic curve in diagnostic test assessment. J Thorac Oncol 2010;5:1315–1316. 20736804 10.1097/JTO.0b013e3181ec173d

[27] Révész D van Kuijk SMJ Mols F . External validation and updating of prediction models for estimating the 1-year risk of low health-related quality of life in colorectal cancer survivors. J Clin Epidemiol 2022;152:127–139. 36220623 10.1016/j.jclinepi.2022.09.019

[28] Osinaike B Ayandipo O Onyeka T Nigerian Surgical Outcomes Study Investigators . Nigerian surgical outcomes - Report of a 7-day prospective cohort study and external validation of the African surgical outcomes study surgical risk calculator. Int J Surg 2019;68:148–156. 31228578 10.1016/j.ijsu.2019.06.003

[29] Van Calster B Wynants L Verbeek JFM . Reporting and interpreting decision curve analysis: a guide for investigators. Eur Urol 2018;74:796–804. 30241973 10.1016/j.eururo.2018.08.038 PMC6261531

[30] Espeso L Coutable A Flaum V . Persistent soiling affects quality of life in children with Hirschsprung’s disease. J Pediatr Gastroenterol Nutr 2020;70:238–242. 31978024 10.1097/MPG.0000000000002564

[31] Saldana Ruiz N Kaiser AM . Fecal incontinence - Challenges and solutions. World J Gastroenterol 2017;23:11–24. 28104977 10.3748/wjg.v23.i1.11 PMC5221273

[32] Teitelbaum DH Drongowski RA Chamberlain JN . Long-term stooling patterns in infants undergoing primary endorectal pull-through for Hirschsprung’s disease. J Pediatr Surg 1997;32:1049–1052. 9247232 10.1016/s0022-3468(97)90397-3

[33] Balachandran VP Gonen M Smith JJ . Nomograms in oncology: more than meets the eye. Lancet Oncol 2015;16:e173–e180. 25846097 10.1016/S1470-2045(14)71116-7 PMC4465353

[34] Toneman MK Krielen P Jaber A . Predicting long-term risk of reoperations following abdominal and pelvic surgery: a nationwide retrospective cohort study. Int J Surg 2023;109:1639–1647. 37042312 10.1097/JS9.0000000000000375 PMC10389206

[35] Schuld J Kreissler-Haag D Remke M . Reduced neorectal capacitance is a more important factor for impaired defecatory function after rectal resection than the anal sphincter pressure. Colorectal Dis 2010;12:193–198. 19183333 10.1111/j.1463-1318.2009.01775.x

[36] Dadhich P Bohl JL Tamburrini R . BioSphincters to treat fecal incontinence in nonhuman primates. Sci Rep 2019;9:18096. 31792260 10.1038/s41598-019-54440-3 PMC6888838

[37] Levitt MA Dickie B Peña A . The Hirschsprungs patient who is soiling after what was considered a “successful” pull-through. Semin Pediatr Surg 2012;21:344–353. 22985840 10.1053/j.sempedsurg.2012.07.009

[38] Bischoff A Frischer J Knod JL . Damaged anal canal as a cause of fecal incontinence after surgical repair for Hirschsprung disease - a preventable and under-reported complication. J Pediatr Surg 2017;52:549–553. 27624566 10.1016/j.jpedsurg.2016.08.027

[39] Travassos DV Bax NM Van der Zee DC . Duhamel procedure: a comparative retrospective study between an open and a laparoscopic technique. Surg Endosc 2007;52:2163–2165. 10.1007/s00464-007-9317-6 PMC2077356 17483999

[40] Demehri FR Dickie BH . Reoperative techniques and management in Hirschsprung disease: a narrative review. Transl Gastroenterol Hepatol 2021;6:42. 34423163 10.21037/tgh-20-224 PMC8343417

[41] Meng X Wang J Zhu T . Long-term outcomes of single-incision laparoscopic technique in Soave procedure compared with heart-shaped anastomosis for Hirschsprung disease. Int J Colorectal Dis 2020;35:1049–1054. 32172319 10.1007/s00384-020-03565-3

[42] Chen F Wei X Chen X . Laparoscopic vs. transabdominal treatment for overflow fecal incontinence due to residual aganglionosis or transition zone pathology in Hirschsprung’s disease reoperation. Front Pediatr 2021;9:600316. 33987148 10.3389/fped.2021.600316 PMC8111174

[43] Meinds RJ van der Steeg AFW Sloots CEJ . Long-term functional outcomes and quality of life in patients with Hirschsprung’s disease. Br J Surg 2019;106:499–507. 30653654 10.1002/bjs.11059 PMC6590339

[44] Menezes M Corbally M Puri P . Long-term results of bowel function after treatment for Hirschsprung’s disease: a 29-year review. Pediatr Surg Int 2006;22:987–990. 17006709 10.1007/s00383-006-1783-8

[45] Grimes DA Schulz KF . Bias and causal associations in observational research. The Lancet 2002;359:248–252. 10.1016/S0140-6736(02)07451-2 11812579

[46] Wang S Zhao M Li T . Stereotactic radiofrequency thermocoagulation and resective surgery for patients with hypothalamic hamartoma. J Neurosurg 2020;134:1019–1026. 32302977 10.3171/2020.2.JNS193423

[47] Bengio Y Grandvalet Y . No unbiased estimator of the variance of k-fold cross-validation. J Mach Learn Res 2004;5:1089–1105.

[48] van Stralen KJ Dekker FW Zoccali C . Confounding. Nephron Clin Pract 2010;116:c143–c147. 20516714 10.1159/000315883

[49] Leacy FP Stuart EA . On the joint use of propensity and prognostic scores in estimation of the average treatment effect on the treated: a simulation study. Stat Med 2014;33:3488–3508. 24151187 10.1002/sim.6030 PMC3995901

[50] Wang S Wang X Zhao M . Long-term efficacy and cognitive effects of voltage-based deep brain stimulation for drug-resistant essential tremor. Clin Neurol Neurosurg 2020;194:105940. 32480294 10.1016/j.clineuro.2020.105940

[51] Wang S Pan J Zhao M . Characteristics, surgical outcomes, and influential factors of epilepsy in Sturge-Weber syndrome. Brain 2022;145:3431–3443. 34932802 10.1093/brain/awab470

